# A New Exercise

**Published:** 1861-05

**Authors:** 


### A NEW EXERCISE.

MANY invalids, sedentary and weakly persons, have found it a serious obstacle in the way of their restoration to health, that they had nothing to do in certain seasons and states of the weather; nothing that they could do which would secure to them the benefit of those bodily activities which have so great an influence not only in working off the old, diseased, decayed, and otherwise useless particles of the system, but in preparing newer, fresher, and more healthy ones to supply their place. To many, an objectless walk, or drive, or ride is a great bore; so is the sawing of wood, and various forms of domestic employments, as they do not exhilerate and interest. Under these circumstances, the parlor-skate, made to go on rollers, attached to the feet and propelled by the same motions as in the common skates for ice, is a most valuable invention. These skates cost from two to five dollars a pair, and are best used on a wooden floor or oil-cloth. Parlor-skating is a most admirable means of strengthening the ankles, than which nothing is more necessary to grace and agility of bodily movements. It is superior to dancing as a mere exercise, because it calls into play a greater number of muscles, brings them into more active exercise and can be done independently: dancing alone is not to be thought of. The variety, grace, and agility of motions obtained by some of the young parlor-skaters is wonderful, as may be seen any day or evening by a visit to Palace Gardens or 446 Broadway.

**PURE MILK FOR CHILDREN.**—It is the general testimony of city physicians that many children become diseased and die, especially in warm weather, from two causes: bad milk and change of kind; using that of one cow to-day and of another to-morrow. Following the example of the veteran editor of the *American Medical Gazette*, Prof. Reese, of the New-York Medical College and Charity Hospital, one of the most accomplished medical scholars on this side the Atlantic, we commend from personal knowledge in a two years' use, the milk furnished by the Rockland County and New-Jersey Milk Association, under the energetic and vigilant superintendence of Mr. S. W. Canfield. This Company has its depot in Rockland county, where there are no distilleries; the milk is taken charge of by the agent while yet warm from farm-house cows, is stirred until cooled; then, being placed in cans surrounded with ice, is forwarded by the Erie Railroad to the city office at No. 146 Tenth street.

Here it is put in cans which are locked and sent direct to customers, who have duplicate keys, thus preventing adulteration by the milkmen. Any family desiring it, can have milk supplied from the same cow during the season, and pure cream at 37½ cents per quart.

**GET THE BEST.**—*The New-York Commercial Advertiser*, one of the oldest and most substantial of the newspapers published in this city, said in its issue for November 19th, of the WORCESTER **PIANO**: "They have stood the test of so many years that they hardly need a word of encomium now. For the better part of a generation, they have been constantly before the public, all of them, old and new, proving by their stability and constancy the skill with which they are constructed. For durability of tone, for evenness and uniformity of work, and for excellence of frames, these pianos may well challenge competition. We know instruments of Mr. Worcester's turn out, that have borne the thummings of fifteen years, and remain as perfect in tone and in build as in their first estate. The number made at this factory is largely increased from year to year." We cordially add the testimony of our own experience to the truth of the *Commercial's* statement, which we find also indorsed in our Southern exchanges.

**BIBLIOGRAPHICAL.**—Dr. Bodenhamer, formerly of Kentucky, now at 854 Broadway, New-York, whom we have represented in these pages as having no superior, if even an equal, in this or any other country, in the treatment of fistulas, fissures, piles, and other diseases of contiguous textures, has just placed himself at the head of American surgery in that department by the issue of a work on the *Malformations of the Rectum and Anus*, which, by the leading medical periodicals and the private testimony of a number of the most distinguished medical professors in the nation, is pronounced "exhaustive;" that it is a work which "must be considered by far the most valuable, if not the only text-book on the subject."

**EYES.**—Prof. Mark Stephenson, of Fifth Avenue, has obtained a high position among the profession in the treatment of all diseases of the eye.

